# Personal Resilience Can Be Well Estimated from Heart Rate Variability and Paralinguistic Features during Human–Robot Conversations

**DOI:** 10.3390/s21175844

**Published:** 2021-08-30

**Authors:** Shin-Min Hsu, Sue-Huei Chen, Tsung-Ren Huang

**Affiliations:** 1 Department of Psychology, National Taiwan University, Taipei 10617, Taiwan; smhsu@mil.psy.ntu.edu.tw (S.-M.H.); shchen@ntu.edu.tw (S.-H.C.); 2 MOST Joint Research Center for AI Technology and All Vista Healthcare, Taipei 10617, Taiwan; 3 MOST AI Biomedical Research Center, Tainan 70101, Taiwan

**Keywords:** automatic personality recognition, human–robot interaction, personal resilience, physiological signals, speech signals

## Abstract

Mental health is as crucial as physical health, but it is underappreciated by mainstream biomedical research and the public. Compared to the use of AI or robots in physical healthcare, the use of AI or robots in mental healthcare is much more limited in number and scope. To date, psychological resilience—the ability to cope with a crisis and quickly return to the pre-crisis state—has been identified as an important predictor of psychological well-being but has not been commonly considered by AI systems (e.g., smart wearable devices) or social robots to personalize services such as emotion coaching. To address the dearth of investigations, the present study explores the possibility of estimating personal resilience using physiological and speech signals measured during human–robot conversations. Specifically, the physiological and speech signals of 32 research participants were recorded while the participants answered a humanoid social robot’s questions about their positive and negative memories about three periods of their lives. The results from machine learning models showed that heart rate variability and paralinguistic features were the overall best predictors of personal resilience. Such predictability of personal resilience can be leveraged by AI and social robots to improve user understanding and has great potential for various mental healthcare applications in the future.

## 1. Introduction

### 1.1. Background and Motivation

People often take good care of their physical health while ignoring their mental health. Correspondingly, while many AI and robot applications for physical healthcare have been developed over the years, those used for mental healthcare applications are limited in number and scope. However, people neglect a crucial fact: like physical illness, mental illness can lead to death [1,2].

Individuals with better protective factors are less likely to suffer from mental health problems, especially when facing traumatic or stressful life events. One of these protective factors is trait resilience [3], which is a positive personality characteristic indicative of one’s adaptability in the face of adversity. For instance, trauma and adversity in childhood may negatively impact stress response systems [4] and contribute to mental disorders such as post-traumatic stress disorder (PTSD) and depression [5,6]. Nevertheless, resilient children can cope well with loss or trauma, not suffer from mental illnesses, and even thrive under such adverse life experiences [7,8].

While an increasing number of psychological studies on resilience indicates a growing interest among researchers in this important topic, there are discrepancies as to how to define and conceptualize personal resilience. In the relevant literature, personal resilience can be discussed as a trait-like capacity, an adaptive process of overcoming adversity, or an outcome of (un)successful adaptation [9]. These different characterizations of personal resilience have led to incongruent resilience measures [10] and hence the difficulty of integrating divergent findings across studies [11]. Among the three characterizations of personal resilience, the trait-like capacity is the only one that precedes adversities from environments [12] and thus can be utilized for early identification and proactive protection of vulnerable populations. Here, the adversities can vary in severity and range from daily hassles to major life events [11], including ostensibly positive and yet stressful life events (e.g., job promotion or marriage) [9].

Despite the values of trait resilience, research on personality computing has mainly focused on estimating the Big Five personality traits [13,14]. For example, many studies have investigated the associations between the Big Five personality traits and physiological [15,16] or linguistic features [17,18,19,20]. By contrast, only a handful of studies have sought biomarkers or behavioral markers of resilience. Moreover, these identified markers are either invasive measures (e.g., neurochemical or immune ones [21]) or linguistic patterns in written narratives (e.g., [22,23]), which are not easily accessible in daily scenarios. To add to the literature on personality computing, the present study set out to investigate whether the psychological resilience of a person can be accurately estimated using simple, cost-effective, and non-invasive procedures.

Specifically, we measured physiological and speech signals to be used as predictors in our resilience-estimating models. These physiological signals can be acquired by wearable devices (e.g., smartwatches or smart bands), and speech signals can be captured during human–human or human–robot conversations. In our resilience-predictive models, the physiology-based predictors included galvanic skin response (GSR), electrocardiograms (ECG), and heart rate variability (HRV); the speech-based predictors encompassed audio and linguistic features. While these physiological signals have been found to correlate with psychological resilience [21], speech markers of resilience, if any, are not yet identified. Therefore, the present study aimed to explore the use of speech signals for predicting personal resilience and compare the predictive powers of these two data sources.

### 1.2. Theoretical Basis

#### 1.2.1. Physiological Signals

GSR, also known as skin conductance or electrodermal activity (EDA), is a biosignal indicating the change in skin conductance resulting from the autonomic activation of sweat glands. Controlled by the sympathetic nervous system, active sweat glands are driven by high-arousal events. When sweat glands are triggered and secrete fluid, the electrical current flow changes. This change leads to a variation in the skin conductance, as often measured by the electrical potential difference between a pair of electrodes placed in the hands or on the feet.

ECG signals are frequently utilized to reveal cardiac conditions. The heart rhythm is influenced by the autonomic nervous system (ANS), which consists of the sympathetic nervous system (SNS) and the parasympathetic nervous system (PNS). While the SNS influences the acceleration and excitation of the heart rate, the influence from the PNS is slow and inhibitory. The average heart rate results from a balance between the SNS and PNS, and the ratio of low- to high-frequency components of the ECG (i.e., LF/HF) indicates such a balance [24].

**Figure 1:**
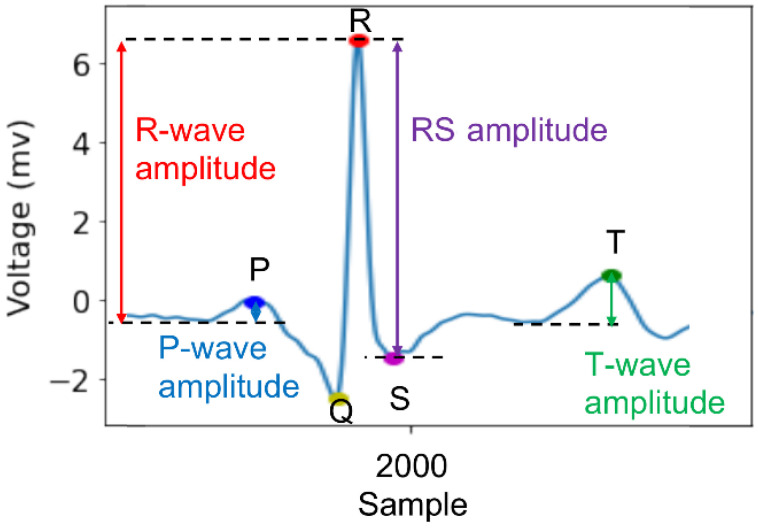
Illustration of ECG waves.

The ECG signal comprises a sequence of positive and negative waves. The first wave, the P wave, is generated by the depolarization of the atria. The following QRS complex represents the depolarization of both ventricles. The last T wave represents ventricle repolarization. As illustrated in Figure 1, the amplitudes of these ECG waves were used in our analyses.

Besides the ECG amplitudes, heart rate variability (HRV) is often computed from ECG signals because it is a strong indicator of one’s cardiac or general health condition. Different indices can be derived from HRV. Time-domain features are extracted based on the variation in the time interval between consecutive heartbeats, such as normal-to-normal (NN) intervals and intervals between adjacent QRS complexes. Frequency-domain features, such as spectrograms, are also frequently examined.

Although few studies have discussed the direct relationship between resilience and physiological signals in healthy individuals, such a relationship may be inferred from the known associations between PTSD and ECG. When faced with traumatic events, less resilient individuals are more likely to suffer from PTSD [25], the ECG characteristics of which include a higher heart rate, lower HF, and higher LF/HF [26,27]. Note, however, that although PTSD is often conceptualized as a *category* of mental disorder, it can be viewed as a spectrum disorder in terms of symptom severity, the nature of the stressor, and responses to trauma [28]. Under such a dimensional conceptualization of PTSD, less resilient individuals that are not diagnosed with full PTSD may, to some extent, also show the aforementioned ECG characteristics, which suggest an elevated sympathetic activity and/or an attenuated parasympathetic activity.

#### 1.2.2. Speech Signals

Speech conveys information through verbal and nonverbal channels. A speaker can communicate meaning not only by verbal contents per se but also by vocal techniques, such as modification of volume, prosody, and intonation (for a review, see [29]). More importantly, while a speaker expresses meaning consciously through these two communication channels, paralinguistic and linguistic features unconsciously expressed during a conversation may reveal the psychological traits or states of the speaker, such as personality or emotional states [30]. Therefore, the current study leveraged paralinguistic and linguistic features extracted from speech signals to estimate psychological resilience.

Paralinguistic features can be further divided into physical and perceptual features [31]. The former is calculated directly from sound waves, including energy function/spectrum and cepstral coefficients; the latter is related to the human perception of the sound, such as loudness, pitch, and rhythm.

As for linguistic features, Linguistic Inquiry and Word Count (LIWC) [32] is a computerized language analysis program widely used to count linguistically or psychologically meaningful words in texts. These words are either content words (e.g., words related to positive emotion or social activities) or function words (e.g., articles, pronouns, and prepositions). While content words deliver semantic meaning, function words partly form linguistic styles [33]. As an example of differences in linguistic style, written texts, relative to spoken texts, are shorter in general but use longer words, more attributive adjectives, and a more varied vocabulary [34]. Therefore, it is unclear how well the linguistic features of resilience found in written texts (e.g., [22,23]) can be used by social robots to estimate personal resilience from speech.

## 2. Materials and Methods

This section presents the materials and methods employed in the study. Section 2.1 describes the study procedure, including the resilience questionnaire, signal recording method, social robot, and task instructions. Section 2.2 introduces the features extracted from physiological and speech signals. Section 2.3 details the feature selection method. Section 2.4 then presents the results from various resilience-estimating models.

### 2.1. Procedure

The whole study took approximately 1.5 h to complete, and each participant was paid $240 New Taiwan Dollars as compensation for research participation. After giving written informed consent of being video- and sensor-recorded, each participant completed a questionnaire that measures personal resilience. Then, the participant was invited by a social robot to recall autobiographical memory with her/his video and physiological signals simultaneously recorded.

#### 2.1.1. Participants

Thirty-five students from National Taiwan University volunteered to participate in the study, but three were excluded from further analysis because they did not follow the instructions. The final sample size was 32 participants (18 females, age range 20–29, M = 22.91, SD = 2.41).

#### 2.1.2. Questionnaires

While there is no “ gold standard” among different measures of resilience, three resilience scales—the Brief Resilience Scale (BRS) [35], the Connor-Davidson Resilience Scale (CD-RISC) [36], and the Resilience Scale for Adults (RSA) [37]—have been found to be psychometrically better than the others [38]. In the present study, we favored the RSA over the other two because the 5-dimensional RSA is a more elaborated measure of resilience than the 1-dimensional BRS and was developed more generally for the healthy population than the more clinically oriented CD-RSIC.

The current study employed the Mandarin Chinese version of the 33-item RSA, which was constructed using a back-translation procedure by the author of this article (S.-H.C.). An exploratory factor analysis of the Mandarin Chinese version partially recovered the five-factor structure of RSA from 29 items: personal strength (6 items), family cohesion (7 items), social resources (8 items), social competence (4 items), and structured style (4 items). The Cronbach’s alphas of the five factors were 0.92, 0.83, 0.87, 0.85, and 0.85, respectively. The overall test-retest correlation of the Mandarin Chinese RSA was 0.89 for an interval of 3–4 weeks.

#### 2.1.3. Signal Recording

We recorded both physiological signals and speech during each human–robot interaction session. Two types of physiological signals—electrocardiography (ECG) and galvanic skin response (GSR)—were measured with a NeXus-10 system (Mind Media BV, Roermond-Herten, Netherlands), which was controlled by the BioTrace+ Software. The ECG signal was measured with an ExG sensor. Following the ECG wrist placement tutorial of the BioTrace+ Software, the ECG electrodes were placed on every participant’s arms. One was placed on the left arm as a reference, one was placed on the lower part of the left arm as the positive channel, and one was attached to the lower part of the right arm as the negative channel. For GSR recording, two electrodes of a GSR sensor were attached separately to the index and ring finger on each participant’s right hand. These signals were monitored on NeXus-10 at a rate of 2048 samples per second. The speech was recorded using Digital Video Recorder H.264 DVR.

#### 2.1.4. Human–Robot Interaction

We used a humanoid social robot—RoBoHoN (Sharp Co., Ltd., Sakai, Japan)—as the conversational agent in the present study. RoBoHoN is a programmable robot with built-in speech-to-text and text-to-speech engines. Nevertheless, at times it misrecognizes spoken words in Mandarin Chinese and speech pauses/endpoints, thereby making inappropriate responses. Because such machine errors might induce negative emotions in the participants and hence become confounding factors, we followed the convention of human–robot studies to adopt a Wizard-of-Oz approach for precisely controlling, via wireless internet, when and what the social robot spoke. Specifically, the social robot functioned as a client who received Remote Procedure Calls (RPCs) of text-based speech commands from a remote computer server. The server offered a web interface for human operators to quickly select predefined sentences (e.g., memory retrieval instructions detailed in the next section) or manually enter on-the-fly texts to be spoken by the social robot. While a participant interacted with the social robot in a well-lit experimental room, the experimenters stayed in a separate room, remotely monitoring the participant’s responses via a real-time video camera and operating the social robot via the web-based control panel. According to our post-study inquiry, all the participants thought the social robot to be under active development and completely autonomous.

#### 2.1.5. Memory Retrieval

To set up the experiment like a casual conversation during a human–robot interaction in healthcare settings, our social robot expressed interest in learning a participant’s personal history and asked each participant to recall memories from his or her life. Six types of memories about social interaction experiences were queried: a positive or a negative memory from either childhood (7–12 years old), adolescence (13–18 years old), or early adulthood (19–24 years old) period. The order of these six queries was randomized for each participant.

The social robot delivered all the memory retrieval instructions, which were adapted from an emotional memory study [39]: “Please describe a memory that related to a positive [negative] experience in a social interaction context during your childhood [adolescence, early adulthood]. The memory should be associated with strong feelings and be something you have thought about many times.” Moreover, each participant was required to recall in steps to separate different cognitive processes, which may be associated with different physiological and/or linguistic features. These steps were (1) memory selection (selecting a specific memory for one minute), (2) memory immersion (immersing oneself in the memory for two minutes), and (3) memory description (describing the memory to the robot without time limit). For the purpose of the current study, only data from the third step were analyzed.

### 2.2. Feature Extraction

#### 2.2.1. Physiological Signals

We applied the first version of the Python package NeuroKit [40] to process ECG signals, including HRV calculation and wave locating. Specifically, ECG signals were first filtered with a Butterworth high-pass filter and transformed into z scores. Then, six HRV features were extracted and directly adopted.

**Figure 2:**
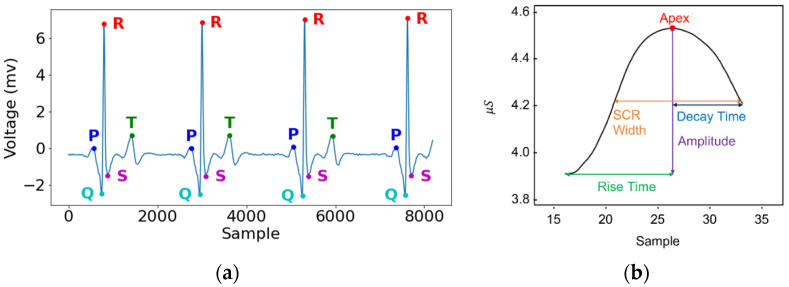
(**a**) Peaks in ECG signals located by the NeuroKit. (**b**) Four GSR features extracted by PySiology.

We calculated five ECG amplitudes on our own to be used as resilience-predicting features because NeuroKit could locate R-, T-, P-, Q-, and S-wave peaks but did not provide the corresponding amplitude values relative to baselines (Figure 2a). Specifically, the baseline voltage preceding a P- or T-wave was averaged from samples 97.65 to 195.31 ms preceding each P or T peak and then subtracted from the corresponding peak voltage to obtain the amplitude of each P- or T-wave. Similarly, the baseline voltage preceding an R-wave was averaged from samples 350 to 300 ms preceding each R peak and then subtracted from the corresponding peak voltage to obtain the amplitude of each R-wave. The QR amplitude is the absolute difference between the Q- and R-peaks; the RS amplitude is the absolute difference between the R- and S-peaks.

Moreover, we used the Python package PySiology [41] to automatically extract five GSR features, as shown in Figure 2b. The rise time is the time during which a GSR rises, from start to apex; the decay time is the time during which a GSR decays to 50% of the amplitude. The GSR amplitude is the value difference between the apex and the start of a GSR. The GSR width is the time interval from 50% amplitude on the increasing side to 50% amplitude on the declining side.

The aforementioned features, together with their standard deviations, then became the final outputs of our feature extraction process. In total, there were five HRV features, ten ECG features, and ten GSR features for later model training and testing, as summarized in Table 1.

#### 2.2.2. Speech Signals

To analyze paralinguistic features in speech, we extracted the Computational Paralinguistics Challenge 2013 (ComParE2013) feature set [42] using the OpenSMILE toolkit [43]. This set contains 6373 acoustic features comprising 65 low-level descriptors (LLDs) and their functionals, as well as five temporal statistics. The LLDs include energy (intensity), spectral, cepstral (MFCC), voice quality (e.g., jitter), shimmer, harmonics-to-noise ratio (HNR), spectral harmonicity, and psychoacoustic spectral sharpness. We have also tested our prediction models with the eGeMAPS feature set [44], which comprises only 88 acoustic features but is not as predictive of resilience as the ComParE2013 features.

To analyze linguistic features in speech, we manually prepared verbatim transcripts of the memories described by the participants and applied CkipTagger [45] to word-segment these Mandarin Chinese transcripts. Then, we adopted the Linguistic Inquiry and Word Count Dictionary for Traditional Chinese (TC-LIWC 2007) to calculate the proportion or relative frequency of 72 psychologically or linguistically meaningful word categories in these transcripts.

### 2.3. Feature Selection

Although mutually correlating features pose the problem of multicollinearity, these mutual correlations do not, in general, affect prediction performances [46]. For example, for an underlying data relationship Y = X_1_ + X_2_ where X_1_ = X_2_ (i.e., a perfect correlation between X_1_ and X_2_), the estimates of β_1_ and β_2_ in the regression model Y = β_1_X_1_ + β_2_X_2_ are unreliable for interpreting the relative importance of X_1_ and X_2_ in predicting Y. However, an infinite number of such regression models can make equally perfect predictions as long as β_1_ + β_2_ = 2. In the current study, when building resilience-predicting models, we were more concerned with the predictive power than the relative importance of features within each modality. Therefore, before model-building, we did not use any dimensionality reduction methods or exclude any features to address the multicollinearity problem.

When constructing multivariate predictive models, we used Pearson’s correlation as a relevance index for univariate feature selection. Specifically, we computed the Pearson’s correlation coefficients between the RSA resilience scores and the features of each modality (i.e., GSR, HRV, ECG, paralinguistic, and linguistic features) and sorted the features according to the absolute values of these coefficients. To achieve the best possible prediction outcome, we treated the number of features for each modality as a model hyperparameter, which was then tuned during the model optimization stage, as detailed in the following section. Finally, each feature was standardized before model training or testing.

### 2.4. Model Training and Testing

For each single-modal resilience-predicting model, the number of selected features was explored with a cap of 6, 10, 10, 100, and 72 features for the HRV, ECG, GSR, paralinguistic, and linguistic modality, respectively. For example, because there were six HRV features before feature selection, six possible sets of HRV features could be constructed after the feature sorting—top six features, top five features, top four features, etc.—and only results from the best-performing HRV feature set were reported.

Two multi-modal feature sets were defined based on their availability in real-life scenarios. The physiological superset combined the best-performing GSR, HRV, and ECG feature sets, as they can be captured by wearable devices; the speech superset combined the best-performing paralinguistic and linguistic feature sets, as they can be obtained from verbal conversations.

For each research participant and each designated feature set, one feature vector could be extracted to characterize each of the participant’s six memory recall episodes (3 life periods × 2 emotional valences). Therefore, there were six feature vectors associated with each participant. To find common patterns in these feature vectors from different memory episodes, all the resilience-predicting models in the current study treated these feature vectors equally as different data samples of the same participant. Moreover, these feature vectors of the same person were grouped to appear only during training or testing but not both for person-level cross-validation.

As for the level of personal resilience, we adopted a classification rather than regression approach when building our resilience-predicting models because of our relatively small number of participants (N = 32). To generate the classification labels, we dichotomized each of the five RSA subscale scores into high vs. low groups based on the median, and the median scores were removed from further analysis.

Four types of machine-learning classifiers were then optimized for comparison: K-Nearest Neighbor (KNN), Logistic Regression (LR), Support Vector Machine (SVM), and Random Forest (RF). These models were trained and tested using 10-fold cross-validation. The hyperparameters of each model were tuned via a grid search to obtain the best average F1-score. For the linear SVM, the best parameter C was determined over [1,11]. The best number of neighbors for KNN was chosen over [1,10]. The best parameter C for logistic regression was chosen among 0.001, 0.01, 0.1, 1, 10, and 100. For the random forest, the number of trees was chosen among 10, 15, 20, 25, and 30. The minimum number of samples required *to split an internal node* was determined over [3,7], and the minimum number of samples required *to be at a leaf node* was explored from 1 to 3.

## 3. Results

Here we present the descriptive statistics of the RSA scores in Section 3.1, the correlations between the RSA scores and personality dimensions as well as physiological/speech features in Section 3.2, and the classification accuracies of the resilience-predicting models in Section 3.3.

Paired sets of results will be presented. The first set corresponds to analyses of data collected during the three recalls of negative memories, whereas the second set corresponds to analyses of all the six recalls of positive and negative memories. The second sets of results are of both theoretical and applied interest. Theoretically, they are verifications of the hypothesis that ostensibly positive events can also be relevant in defining resilience [9]. For applications, it will be convenient to estimate one’s resilience level regardless of narrative contexts.

### 3.1. Personal Resilience

As mentioned in Section 2.4, for each resilience dimension, the 32 participants were divided into high- and low-score groups. The total, median, mean, and standard deviation of the RSA scores are summarized in Table 2 for both the high- and low-score groups.

### 3.2. Correlational Analysis

**Figure 3:**
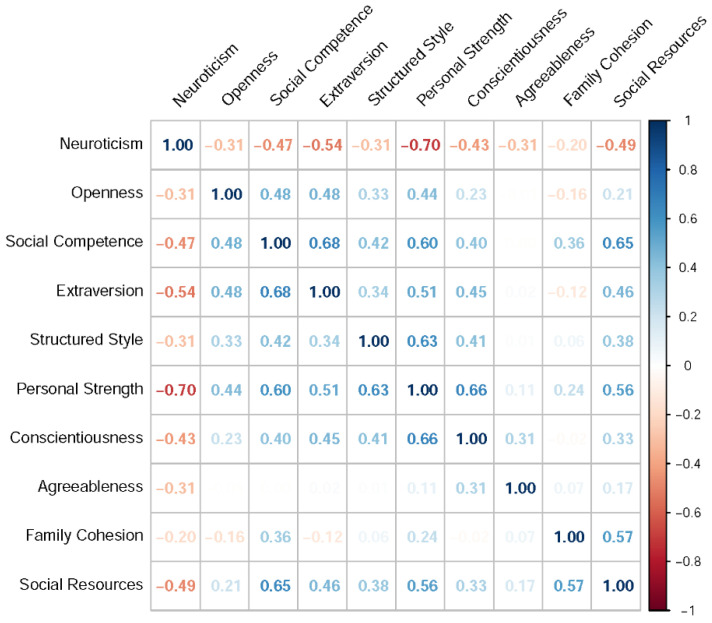
The correlations between each RSA score and each personality dimension.

**Figure 4:**
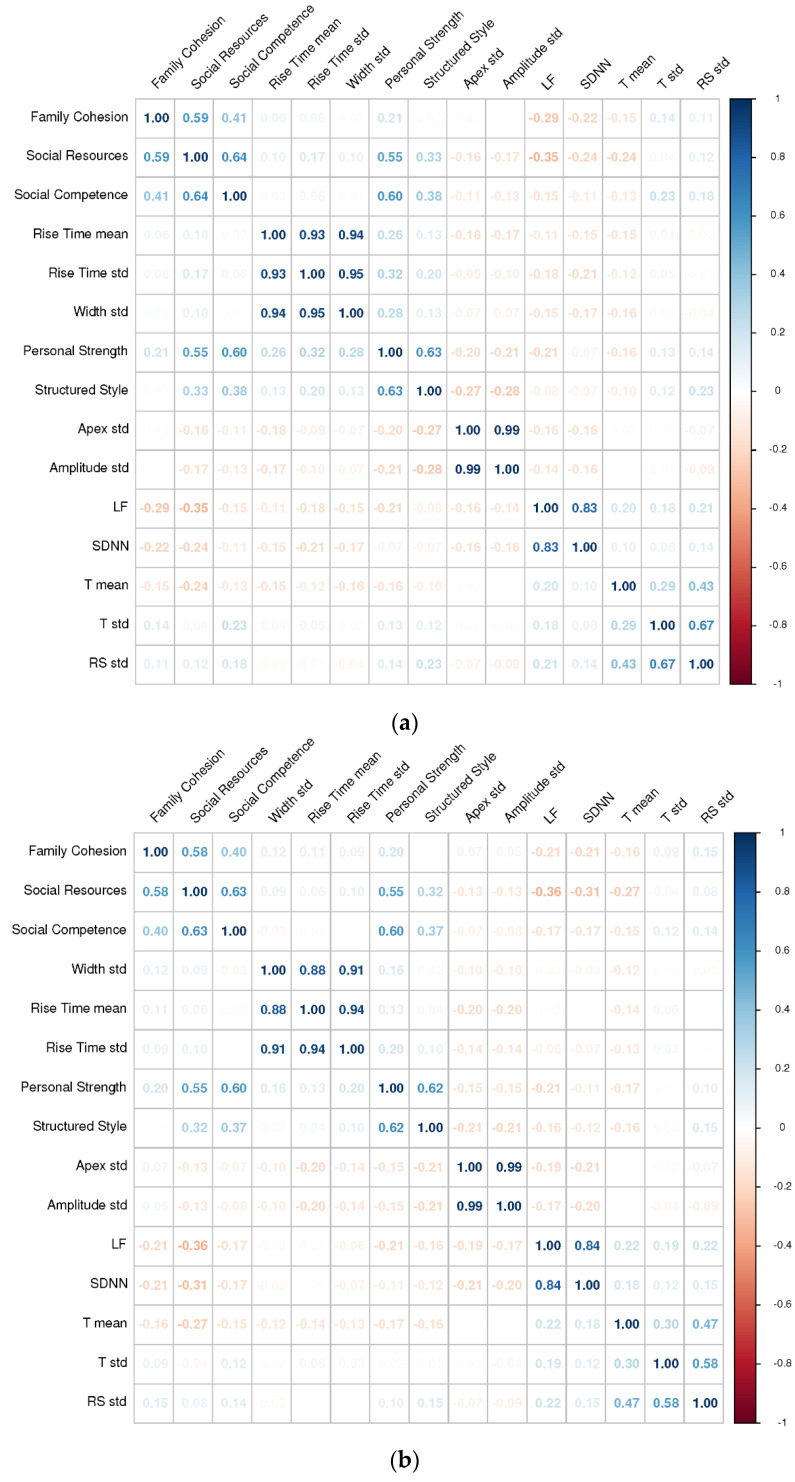
(**a**). The correlations between each RSA score and each physiological feature (*only negative memories*). Physiological features that were not significantly correlated with any RSA dimensions (i.e., *p* > 0.05) are excluded from the plot. (**b**). The correlations between each RSA score and each physiological feature (*positive and negative memories*). Physiological features that were not significantly correlated with any RSA dimensions (i.e., *p* > 0.05) are excluded from the plot.

**Figure 5:**
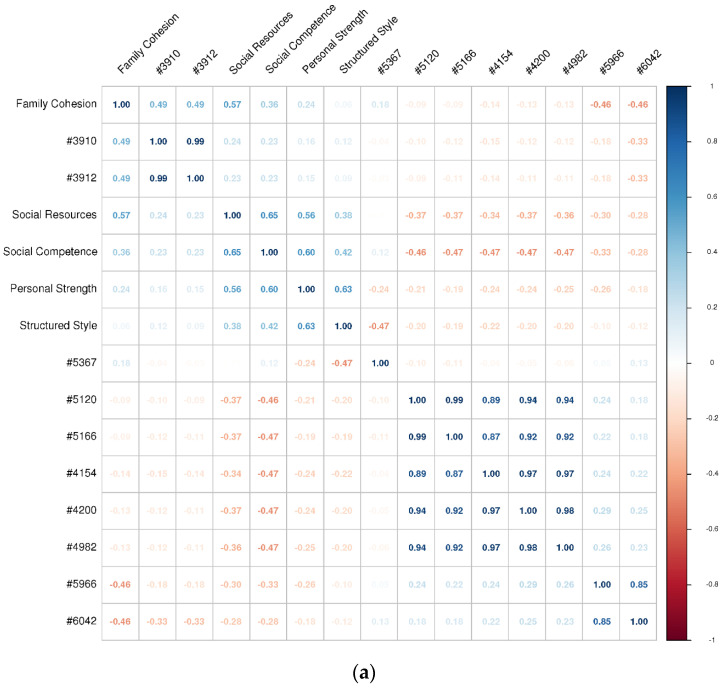

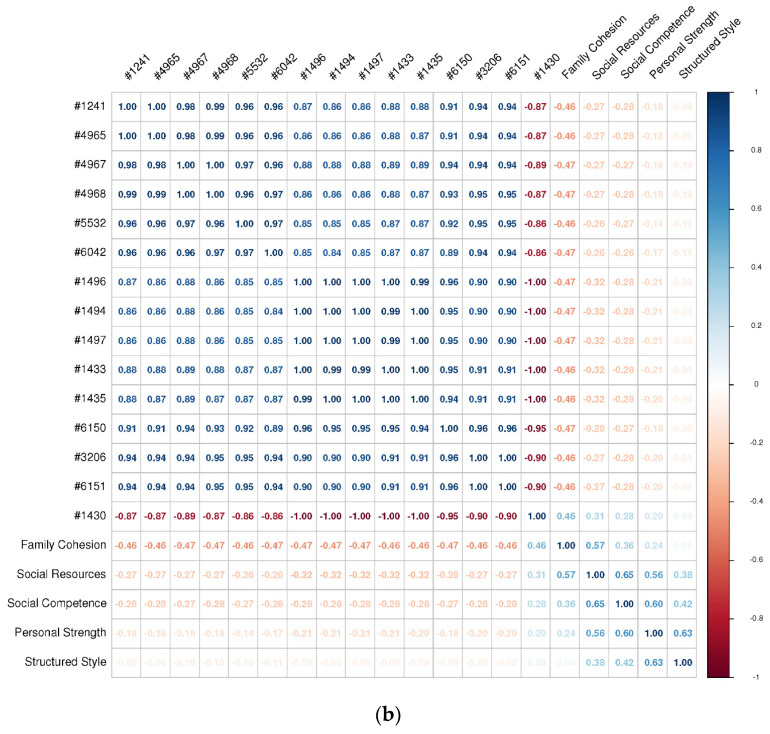
(**a**). The correlations between each RSA score and each ComParE2013 paralinguistic feature *(only negative memories).* Because there were many statistically significant correlations (*p* < 0.05), only paralinguistic features with at least one |r| > 0.46 were included in this plot. **#3910** = F0final_sma_de_quartile3; **#3912** = F0final_sma_de_iqr2−3; **#5367** = mfcc_sma[9]_linregc2; **#5120** = pcm_fftMag_spectralSlope_sma_centroid; **#5166** = pcm_fftMag_spectralHarmonicity_sma_centroid; **#4154** = audspec_lengthL1norm_sma_centroid; **#4200** = pcm_RMSenergy_sma_centroid; **#4982** = pcm_fftMag_spectralFlux_sma_centroid; **#5966** = pcm_fftMag_fband1000−4000_sma_de_stddevRisingSlope; **#6042** = pcm_fftMag_spectralFlux_sma_de_meanFallingSlope. (**b**). The correlations between each RSA score and each ComParE2013 paralinguistic feature *(positive & negative memories).* Because there were many statistically significant correlations (*p* < 0.05), only paralinguistic features with at least one |r| > 0.46 were included in this plot. **#1241** = pcm_fftMag_spectralFlux_sma_range; **#4965** = pcm_fftMag_spectralFlux_sma_peakRangeAbs; **#4967** = pcm_fftMag_spectralFlux_sma_peakMeanAbs; **#4968** = pcm_fftMag_spectralFlux_sma_peakMeanMeanDis; **#5532** = pcm_RMSenergy_sma_de_meanFallingSlope; **#6042** = pcm_fftMag_spectralFlux_sma_de_meanFallingSlope; **#1496** = pcm_fftMag_spectralHarmonicity_sma_iqr2−3; **#1494** = pcm_fftMag_spectralHarmonicity_sma_quartile3; **#1497** = pcm_fftMag_spectralHarmonicity_sma_iqr1−3; **#1433** = pcm_fftMag_spectralSlope_sma_iqr1−2; **#1435** = pcm_fftMag_spectralSlope_sma_iqr1−3; **#6150** = pcm_fftMag_spectralHarmonicity_sma_de_posamean; **#3206** = pcm_fftMag_spectralHarmonicity_sma_de_stddev; **#6151** = pcm_fftMag_spectralHarmonicity_sma_de_rqmean; **#1430** = pcm_fftMag_spectralSlope_sma_quartile1.

**Figure 6:**
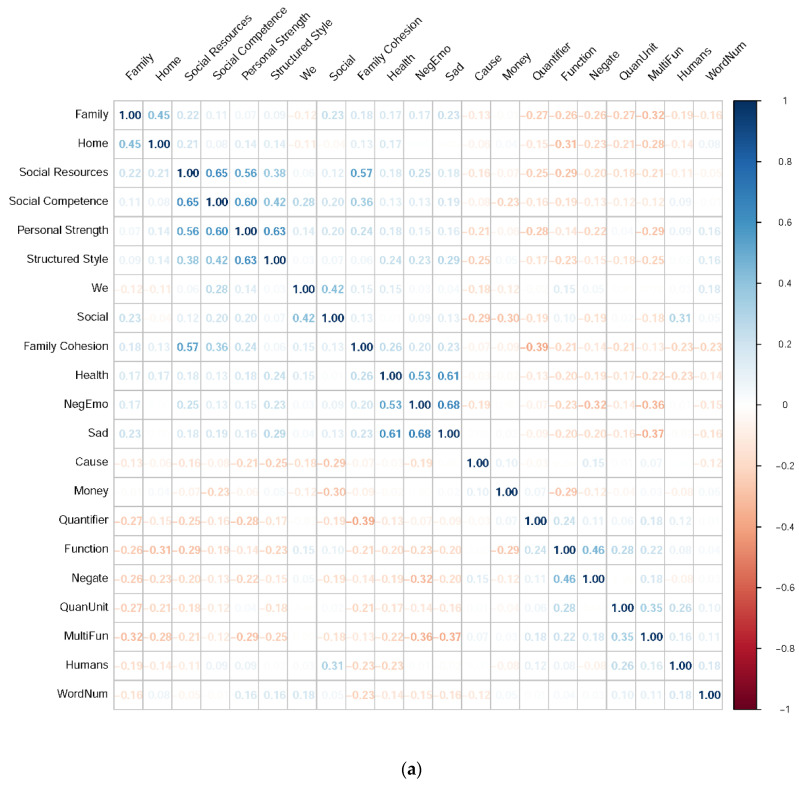

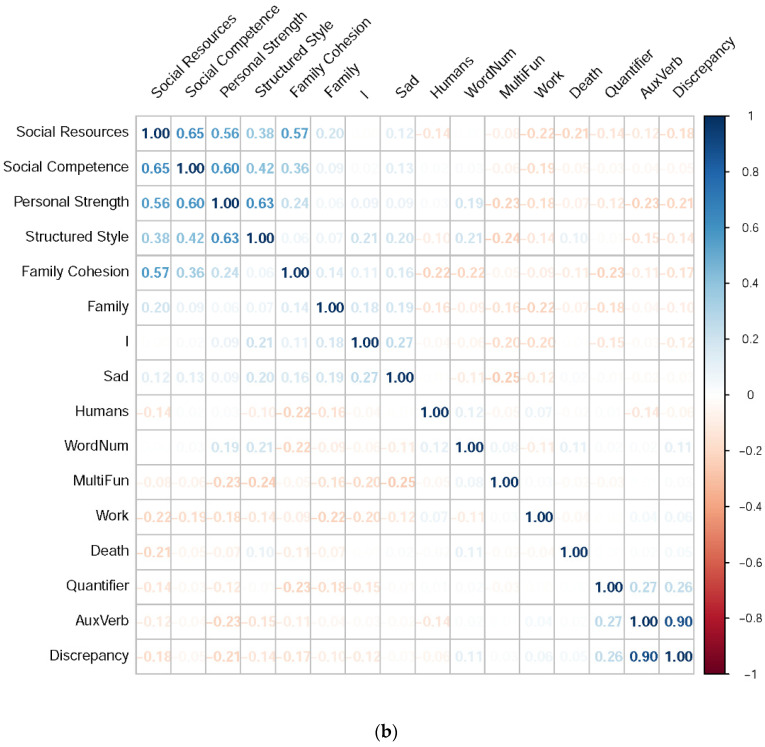
(**a**). The correlations between each RSA score and each TC-LIWC linguistic feature *(only negative memories).* Because there were many statistically significant correlations (*p* < 0.05), only linguistic features with at least one |r| > 0.2 were included in this plot. **MultiFun** = multifunction words; **NegEmo** = negative emotion; **QuantUnit** = quantity unit; **WordNum** = number of words. (**b**). The correlations between each RSA score and each TC-LIWC linguistic feature *(positive and negative memories).* Because there were many statistically significant correlations (*p* < 0.05), only linguistic features with at least one |r| > 0.2 were included in this plot. **AuxVerb** = auxiliary verbs; **MultiFun** = multifunction words; **NegEmo** = negative emotion; **WordNum** = number of words.

The Pearson correlation coefficients between each RSA score and each resilience-predicting feature are summarized in Figure 3, Figure 4, Figure 5 and Figure 6. These correlation matrices were sorted based on the results of hierarchical clustering so that more correlated variables were arranged closer to each other in the matrices. Such matrix sorting helps to reveal the structures of correlations between RSA scores and their potential predictors in this explorative study.

#### 3.2.1. Big-Five Personality

The correlations between RSA and personality scores are summarized in Figure 3. These results are consistent with the previous finding that RSA directly measures a well-adjusted personality profile [47]. For example, *social competence* was positively correlated with extraversion and openness, *structured style* was positively correlated with conscientiousness, and *personal strength* was negatively correlated with neuroticism. In other words, a person high in RSA scores is likely to be an emotionally stable extravert who is also conscientious and open to new experiences.

Note also from Figure 3 that the five RSA dimensions were not independent of each other. Specifically, dimensions related to personal characteristics—*social competence*, *structured style*, and *personal strength*—were mutually associated as a group, whereas dimensions related to environmental factors—*family cohesion* and *social resources*—were covaried as another group. This correlation structure reflects the fact that RSA was designed to measure protective factors of resilience along both intrapersonal and interpersonal dimensions [37].

#### 3.2.2. Physiological Features

As shown in Figure 4a,b, among all the physiological features, two HRV features—the low-frequency component (i.e., LF) and the standard deviation of the NN intervals (i.e., SDNN)—are most associated with all the five resilience dimensions, especially *social resources*. Here, the higher LF and SDNN in less resilient individuals might indicate a higher sympathetic and/or low parasympathetic nervous activity, as suggested by previous studies [26,27,48] under the dimensional conceptualization of PTSD.

Among all the ECG features, the mean amplitude of the T-wave (i.e., T mean) appears to be most indicative of resilience, particularly *social resources*. The negative correlation between T mean and resilience is in line with the finding that the T-wave amplitude is positively associated with psychological stress [49], which is, however, less experienced by more resilient individuals [9].

Among all the GSR features, the variability of rise time, apex, and amplitude (i.e., RT std, AP std, and AM std) are most indictive of resilience, particularly *personal strength* and *structured style*. These findings are consistent with a body of literature showing GSR to reflect one’s emotionality. For example, emotionally evocative pictures can induce larger and more prolonged GSRs in more neurotic individuals [50]. It is likely that less resilient individuals may also show such GSR characteristics and their associated signatures in variability. Specifically, we found resilience to positively correlate with RT std but negatively correlate with the AP/AM std.

#### 3.2.3. Paralinguistic Features

Among all the 6373 acoustic features, only the ones that were most correlated with RSA scores are reported in Figure 5a,b. Overall, the most resilience-relevant features are spectral features, including ones related to the fundamental frequency (F0*), auditory spectrum coefficients (audspec*), Mel Frequency Cepstral Coefficients (mfcc*), spectral slope (pcm_fftMag_spectralSlope*), spectral flux (pcm_fftMag_spectralFlux*), and spectral harmonicity (pcm_fftMag_spectralHarmonicity*). The correlations between these spectral features with RSA scores may have to do with individuals’ emotional styles unconsciously expressed through speech. In the literature of affective computing, it has been found that F0 and spectral distribution are important cues of affective states [51], and MFCC and spectral flux are particularly informative of emotional valence [44]. Given that emotions are regulated by resilience [9,52], resilience dimensions can then be linked with these spectral features.

#### 3.2.4. Linguistic Features

Among all the 72 TC-LIWC categories, only word categories most correlated with RSA scores are reported in Figure 6a,b. The detailed descriptions and exemplars of each category can be found elsewhere [53]. Note first from the figures that hierarchically organized TC-LIWC categories are not independent of each other, and the correlations among TC-LIWC categories partially reflect their overlapping vocabularies (e.g., sad and negative emotion, we and social, auxiliary verbs and discrepancy words, etc.) and partially reflect the structures among their corresponding psychological dimensions. Secondly, while only a few physiological features were correlated with resilience, several linguistic features covaried with different dimensions of resilience. These results echo the findings that LIWC is generally sensitive to detect individual differences in terms of attentional focus, thinking styles, emotionality, and social relationships [54].

While some of the RSA-LIWC correlations are sensible (e.g., *Family Cohesion* and family words, *Social Competence*/*Resources* and social words, etc.), negative emotion words (e.g., sad) are unexpectedly used more by highly resilient individuals in the present study. Although past studies have often shown that resilient people thrive through positive emotions [52,55], resilient people do respond to negative events initially with negative affect [52]. Empirical evidence further suggests that trait resilience is not well characterized by unconditional positive emotions but by the ability to flexibly switch emotional responses to match the environmental demands [56]. Other studies that analyzed written texts (e.g., diaries in [22]) might observe more frequent use of positive emotions by highly resilient individuals as a result of their positive appraisal of events [9]. By contrast, our memory immersion procedure might vividly reactivate negative memories of the participants and thereby accentuate the characteristics of their emotional reactivity rather than cognitive appraisal. Future studies can further investigate such a possibility of procedural/contextual influences on linguistic features in association with resilience.

### 3.3. Classification Results

Table 3 and Table 4 present the mean F1-scores of resilience predictions using different modalities with different types of classifiers. Overall, different classifiers led to similar prediction performances, whereas a relatively large variability in performance was observed across different combinations of a resilience dimension and its predicting modality. For example, the physiological features (i.e., ECG, HRV, and GSR) were poor predictors of *social competence* but strong predictors of *social resources* during the recall of negative memories. By contrast, speech features (i.e., paralinguistic and linguistic features) were also poor predictors of *social competence* but strong predictors of *structured style* during the recall of negative memories.

Among the single modalities, the HRV and acoustic features are overall the best predictors of resilience across narrative contexts and resilience dimensions. This finding has been hinted at by the relatively large correlations between the HRV/acoustic features and the RSA scores (Figure 4a,b and Figure 5a,b). Note also that predictions that leverage features from multiple modalities were not necessarily better than those using HRV/acoustic features. In the case of physiological signals, adding the poorly performing ECG and GSR features into the augmented feature set might actually decrease the signal-to-noise ratio in the data. In the case of speech signals, the 6373 acoustic features outnumbered the 72 TC-LIWC features in the augmented feature set and might shadow the additional information brought by TC-LIWC features.

## 4. Discussion & Conclusions

The present study confirms the possibility of estimating personal resilience from speech and physiological signals. Our binary classification of personal resilience successfully achieved F1-scores as high as 0.86 in the cases of predicting *social resources* by the HRV features during the recall of negative memories and predicting *structured style* by the paralinguistic features during the recall of positive and negative memories. Our results suggest that the HRV and paralinguistic features are the best predictors of resilience.

For healthcare applications, the HRV data can be conveniently collected from wearable devices, and the paralinguistic features can be collected by a social robot during human–human or human–robot conversations to estimate personal resilience. Compared to the extraction of linguistic features, extraction of paralinguistic features does not require automatic speech recognition (ASR), which can be erroneous at times. Therefore, resilience estimation is expected to be more accurate and robust by using the paralinguistic than the linguistic features.

Note that the HRV and paralinguistic features led to comparable prediction performances across positive and negative contexts. It is an important discovery that trait resilience can be well-estimated regardless of narrative contexts. Theoretically, this finding confirms the hypothesis that ostensibly positive events are also relevant in defining resilience [9], which extends the scope of personal resilience. Practically, it is then not required for resilience estimation to actively set up or passively wait for a negative context in which an individual can manifest her/his resilience or lack thereof.

In summary, our research adds to the literature on psychological resilience and personality computing by showing the predictability of personal resilience across different contexts. Automatic assessment of resilience can assist healthcare providers and AI systems/robots in identifying and planning interventions for less resilient individuals. For example, medical practitioners or social robots can be informed to avoid putting too much pressure on individuals low in resilience during interactions, and this vulnerable population can be taught suitable coping strategies for reducing chronic stress.

To conclude, we hope that this explorative study will arouse the interest of personality-computing and social robotics researchers to further advance the automatic recognition of personal resilience. During the COVID-19 pandemic, social distancing prevents people from making social connections, causing loneliness and social isolation. Quarantine may have negative impacts on both physical and mental health and lead to severe consequences, such as a higher rate of suicide. Previous research suggests that resilience can reduce suicidality risk and play a critical role in suicide prevention [57]. Therefore, the predictability of personal resilience can be leveraged to improve one’s psychological well-being and has great potential for various mental healthcare applications in the future.

## Figures and Tables

**Table 1:** HRV, ECG, and GSR features.

HRV Features		ECG Features	GSR Features
Features	Meaning	Features	Features
meanNN	The mean of normal-to-normal (NN) intervals	P mean & std R mean & std T mean & std QR mean & std RS mean & std	Rise Time mean & std Amplitude mean & std Apex mean & std Decay Time mean & std Width mean & std
SDNN	The standard deviation of the NN intervals		
RMSSD	The root mean square of the RR intervals		
LF	Power in the low-frequency range (0.04–0.15 Hz)		
HF	Power in the high-frequency range (0.15–0.40 Hz)		
LF/HF	The ratio of LF to HF		

**Table 2:** Summary statistics of the RSA scores (N = 32).

Resilience	PS (M ± SD)	FC (M ± SD)	SR (M ± SD)	SC (M ± SD)	SS (M ± SD)
**Median/Total**	30/42	35.5/49	44/56	17.5/28	20/28
**High Group**	34.00 ± 3.20	39.56 ± 3.12	48.44 ± 3.33	22.88 ± 2.25	23.25 ± 2.86
**Low Group**	21.40 ± 5.26	28.44 ± 4.82	36.93 ± 3.91	14.06 ± 2.29	14.58 ± 2.50

Note. PS = Personal Strength, FC = Family Cohesion, SR = Social Resources, SC = Social Competence, SS = Structured Style.

**Table 3:** Results of Resilience Estimation *(only negative memories)*.

Resilience Dimension	Model	Single Modality					Multiple Modalities	
		GSR	ECG	HRV	Acoustic	LIWC	Physiology	Speech
Personal Strength	KNN	0.61	0.70	0.75	0.69	0.60	0.65	0.65
	LR	0.59	0.68	***0.76***	*0.71*	*0.66*	0.68	***0.71***
	SVC	*0.66*	*0.72*	0.72	0.69	0.63	*0.70*	0.67
	RF	*0.66*	*0.72*	0.72	0.67	0.63	*0.70*	0.64
Family Cohesion	KNN	*0.63*	0.64	*0.70*	***0.75***	0.69	*0.68*	***0.74***
	LR	0.42	*0.68*	0.69	0.71	0.68	0.57	0.69
	SVC	0.60	0.63	0.69	0.72	*0.70*	0.55	0.68
	RF	0.60	0.63	0.69	0.66	0.68	0.63	0.60
Social Resources	KNN	0.69	*0.76*	***0.86***	0.64	0.69	***0.82***	*0.68*
	LR	0.60	0.71	0.82	0.65	0.64	0.78	0.53
	SVC	*0.73*	0.73	0.84	*0.67*	0.65	0.79	0.63
	RF	*0.73*	0.73	0.84	0.65	*0.73*	0.81	0.62
Social Competence	KNN	0.42	*0.54*	*0.51*	*0.65*	0.66	0.45	***0.66***
	LR	*0.49*	0.46	0.48	0.58	0.67	0.49	***0.66***
	SVC	0.46	0.50	0.49	0.60	***0.70***	0.47	0.63
	RF	*0.49*	0.46	0.48	0.62	0.68	*0.50*	0.63
Structured Style	KNN	0.75	0.78	0.68	0.77	0.72	0.77	0.75
	LR	*0.76*	*0.80*	***0.81***	0.79	0.73	0.74	0.76
	SVC	0.72	0.74	0.69	***0.81***	0.72	0.77	***0.79***
	RF	0.72	0.74	0.69	0.79	*0.76*	*0.79*	0.78

Note. For each resilience dimension, the best F1-scores for each modality are highlighted in italics, and the best F1-scores across modalities are further highlighted in bold.

**Table 4:** Results of Resilience Estimation (*positive and negative memories*).

Resilience Dimension	Model	Single Modality					Multiple Modalities	
		GSR	ECG	HRV	Acoustic	LIWC	Physiology	Speech
Personal Strength	KNN	*0.57*	*0.63*	***0.77***	0.62	0.64	*0.48*	0.64
	LR	0.54	*0.63*	0.76	*0.70*	*0.66*	0.47	***0.70***
	SVC	0.56	*0.63*	0.74	0.54	0.64	0.45	0.63
	RF	0.56	*0.63*	0.74	0.64	*0.66*	0.44	0.67
Family Cohesion	KNN	*0.56*	0.63	0.65	0.56	*0.58*	0.50	0.56
	LR	0.43	***0.68***	***0.68***	***0.68***	0.55	0.43	***0.66***
	SVC	0.52	0.63	0.67	0.59	0.54	0.38	0.56
	RF	0.52	0.63	0.67	0.58	0.52	*0.63*	0.53
Social Resources	KNN	*0.60*	0.72	***0.85***	0.64	0.63	0.51	0.66
	LR	0.53	0.66	0.80	*0.67*	0.65	0.47	***0.68***
	SVC	0.58	*0.73*	0.84	0.62	*0.68*	0.45	0.50
	RF	0.58	*0.73*	0.84	0.63	0.62	*0.54*	0.60
Social Competence	KNN	0.37	0.43	*0.57*	***0.69***	*0.58*	*0.41*	***0.71***
	LR	*0.48*	*0.46*	0.53	***0.69***	0.57	0.30	***0.71***
	SVC	0.37	0.43	0.55	***0.69***	*0.58*	0.28	0.65
	RF	*0.48*	*0.46*	0.53	0.65	*0.58*	0.36	0.64
Structured Style	KNN	0.72	0.68	0.69	0.81	0.73	0.67	0.79
	LR	0.72	*0.79*	*0.82*	***0.86***	*0.74*	0.58	***0.86***
	SVC	*0.74*	0.69	0.69	0.81	0.72	0.69	0.75
	RF	*0.74*	0.69	0.69	0.78	0.73	*0.71*	0.76

Note. For each resilience dimension, the best F1-scores for each modality are highlighted in italics, and the best F1-scores across modalities are further highlighted in bold.

## Data Availability

The data presented in this study are available on request from the corresponding author. The data are not publicly available due to privacy or ethical restrictions.
